# Assessment of aortomesenteric distance and mesenteric and retroperitoneal adipose tissue thickness in genetic forms of lipodystrophy

**DOI:** 10.1007/s40618-024-02429-9

**Published:** 2024-10-01

**Authors:** Mehmet Cagri Unal, Furkan Uncuoglu, Gokcen Gungor Semiz, Mehmet Emin Arayici, Serkan Yener, Canan Altay, Baris Akinci

**Affiliations:** 1 Department of Endocrinology and Metabolism, Dokuz Eylul University, Izmir, Turkey; 2 Department of Radiology, Dokuz Eylul University, Izmir, Turkey; 3 Department of Biostatistics and Medical Informatics, Dokuz Eylul University, Izmir, Turkey; 4 DEPARK, Dokuz Eylul University, Izmir, 35340 Turkey; 5 Izmir Biomedicine and Genome Center, Izmir, Turkey

**Keywords:** Lipodystrophy, Visceral adipose tissue, Mesenteric adipose tissue, Aortomesenteric distance

## Abstract

**Introduction:**

Lipodystrophy is a rare disease characterized by the loss of adipose tissue. Visceral adipose tissue loss in certain forms of lipodystrophy may affect the amount of mesenteric fat.

**Method:**

We studied visceral adipose tissue by measuring the thickness of mesenteric and retroperitoneal adipose tissue and the aortomesenteric (AOM) distance in patients with genetic forms of lipodystrophy (*n* = 48; 7 males; 41 females; mean age 39.1 ± 11.9 years; 19 with congenital generalized lipodystrophy [CGL], and 29 with familial partial lipodystrophy [FPLD]). An age- and gender-matched control group with a ratio of 1:2 was generated.

**Results:**

Patients with CGL had severely depleted mesenteric adipose tissue (2.0 [IQR: 1.5–3.5] mm vs. 18.8 [IQR: 4.4–42.2] mm in FPLD, *P* < .001; 30.3 [IQR: 13.9–46.6] mm in controls, *P* < .001) and retroperitoneal adipose tissue (1.3 [IQR: 0.0–5.3] mm vs. 33.7 [IQR: 21.6–42.1] mm in FPLD, *P* < .001; 29.7 [IQR: 23.1–36.7] mm in controls, *P* < .001). The AOM distance was shorter in patients with CGL (8.1 [IQR: 6.0–10.8] mm) compared to patients with FPLD (vs. 13.0 [IQR: 8.8–18.1] mm; *P* = .023) and controls (vs. 11.3 [IQR: 8.4–15.5] mm, *P* = .016). Leptin levels were positively correlated with AOM distance in lipodystrophy (*r* = .513, *P* < .001). Multivariate linear regression analysis identified body mass index as a significant predictor of AOM distance (data controlled for age and sex; beta = 0.537, 95% CI: 0.277–0.798, *P* < .001). Twelve of 19 patients (63%) with CGL had an AOM distance of < 10 mm, a risk factor that may predispose patients to developing superior mesenteric artery syndrome.

**Conclusion:**

CGL is associated with a severe loss of mesenteric adipose tissue, which leads to a narrowing of the space between the superior mesenteric artery and the aorta.

## Introduction

Superior mesenteric artery (SMA) syndrome is a rare cause of duodenal outlet obstruction characterized by compression of the third section of the duodenum as a result of space shrinking between the SMA and aorta due to the loss of the intervening mesenteric lipid pad [1]. SMA syndrome has been previously associated with rapid weight loss caused by multiple etiologies that can narrow the aortomesenteric (AOM) angle [2–8]. Congenital or acquired anatomical anomalies may also contribute to the development of this rare syndrome [9]. Patients with SMA syndrome may exhibit acute or more gradually developing symptoms indicating proximal small bowel obstruction. While those with more advanced obstruction may experience severe nausea, bilious emesis, and weight loss, patients with minor obstruction may present with milder symptoms, such as postprandial epigastric pain, early satiety, and gastroesophageal reflux [10].

Lipodystrophy is a rare condition characterized by the loss of adipose tissue that affects near-total (generalized) or certain parts (partial) of the body, which both can be inherited or acquired [11, 12]. Adipose tissue loss in lipodystrophy can lead to metabolic abnormalities associated with insulin resistance [13] and can also affect the amount of visceral and mesenteric fat, which can potentially narrow the space between the superior mesenteric artery and aorta, potentially causing compression of the third portion of the duodenum. SMA syndrome is mainly developed due to events that can cause the loss of intervening mesenteric fat (e.g., rapid weight loss, malnutrition, eating disorders, gastric bypass surgery, malignancies, and inflammatory bowel disease).

Abdominal pain is a relatively commonly reported symptom in patients with lipodystrophy and is usually attributed to episodes of pancreatitis [14]; however, it can be caused by several other pathologies, such as severe hepatomegaly, infections, and intestinal dysmotility. Whether the loss of mesenteric fat in lipodystrophy leads to shortening of the AOM distance, a risk factor for SMA syndrome development, or SMA symptoms is unknown. In this study, we measured the AOM distance in patients with genetic forms of lipodystrophy that can affect the amount of mesenteric fat pad. Patients were also questioned about the symptoms of SMA syndrome.

## Materials and methods

### Subjects

Forty-eight patients (41 females and 7 males) with genetic forms of lipodystrophy were included in the study. The mean age was 38.3 ± 12.5 years (median age, 35.0 years [IQR: 29.0–46.5]). Nineteen of them had congenital generalized lipodystrophy (CGL) and 29 had familial partial lipodystrophy (FPLD). An age- and gender-matched control group (*n* = 96; 1:2) was generated from a cohort of subjects with hormone-inactive adrenal incidentalomas. Those with malignancies, adrenal carcinoma, and hormone-producing adrenal adenomas, including overt and subclinical Cushing syndrome, pheochromocytoma, and hyperaldosteronism, were excluded.

The ethics committee of Dokuz Eylul University approved the study protocol (approval ID: 2022/04–07). All patients provided written consent for genetic testing. No sponsor is involved in the study design, data collection, analysis, report writing, or the decision to submit for publication.

### MRI acquisition

Magnetic resonance imaging (MRI) was performed at Dokuz Eylul University between 2013 and 2022. The MRI protocol comprised an axial T1-weighted turbo spin-echo image with the following parameters: repetition time/echo time, 396/17; flip angle, 90°; echo train length, 6; field of view, 50 × 180 cm; and slice thickness, 5 mm. The scans were part of a whole-body MRI ordered to assess fat distribution. A single-slice axial MR image at the aortomesenteric level was selected from these previously obtained whole-body or abdominal MRIs. To ascertain visceral fat in subjects, we employed a method of measuring the distance between the transversalis facia and the superior mesenteric artery (TF-SMA) at the level of the umbilicus, which represents the thickness of the mesenteric adipose tissue. To demonstrate the thickness of retroperitoneal adipose tissue, the distance from the end of the right renal lower pole to the transversalis facia (TF-RR) was measured (perirenal and posterior pararenal space thickness). We only included the fat tissue signal in these measurements and excluded all bowel segments in the region from the measured value. The 1.5-T MRI system (Gyroscan Achieva, release 8.1; Philips Medical Systems) equipped with a 6-multichannel body coil was used. Using the same methodology, a single-slice axial MRI was used to measure the AOM distance at the aortomesenteric level obtained from abdominal MRI scans in the control group.

### Symptom screening and medical chart review

A questionnaire specifically developed by the investigators was used to assess symptoms indicative of SMA syndrome. This questionnaire was designed based on the most common gastrointestinal (GI) symptoms associated with SMA syndrome. In addition, medical charts were thoroughly reviewed to identify symptoms potentially attributed to SMA syndrome. Specifically, all instances where patients presented with abdominal pain and were admitted to the endocrinology clinic, emergency room, or hospitalized were scrutinized.

To further investigate potential patients with SMA syndrome, computed tomography (CT) and/or Doppler ultrasonography were used to measure the AOM angle. This was performed if clinical suspicion arose when an AOM distance of < 10 mm was determined in MRI.

### Statistical analysis

The lipodystrophy group was stratified into two subgroups: CGL and FPLD. To establish a comparable control, an age- and gender-matched group was generated with a 1:2 ratio from a cohort of individuals with hormone-inactive adrenal incidentalomas. Propensity score matching was employed to minimize selection bias when statistically comparing clinical outcomes between patients with and without lipodystrophy. Matching was performed using a caliper set twice that of the control group compared with the lipodystrophy group. The Statistical Package for the Social Sciences (SPSS, IBM Corp) version 29.0 for Windows was used for all statistical analyses. The Shapiro–Wilk and Kolmogorov–Smirnov tests were performed to assess the normal distribution of variables. Continuous data were expressed as medians (interquartile ranges [IQR]: 25th–75th), and categorical parameters were analyzed using the chi-square test. The Mann–Whitney U-test was employed to compare non-normally distributed continuous parameters. A *P* value of 0.05 or lower was considered statistically significant.

## Results

Table 1 summarizes the baseline characteristics of patients and controls. Among the 19 patients with CGL, 12 had generalized fat loss caused by pathogenic variants in acyltransferase 1-acylglycerol-3-phosphate O-acyltransferase 2 (*AGPAT2*), 4 had seipin (*BSCL2*), 2 had caveolae associated protein 1 (*CAVIN-1*), and 1 had lamin A/C (*LMNA*) (Table 2).

**Table 1:** Clinical and laboratory features and aortomesenteric distance between patients with lipodystrophy and controls

	Lipodystrophy vs. Control				CGL vs. Control			CGL vs. FPLD	
	Lipodystrophy (*n* = 48)	Control (*n* = 96)	*P* value		CGL (*n* = 19)	*P* value		FPLD (*n* = 29)	*P* value
Age, years	35 (29–46)	41 (34–48)	0.066		29 (25–35)	< 0.001		44 (33–50)	< 0.001
BMI, kg/m^2^	22.1 (19.5–26.1)	25.2 (23.3–27.5)	< 0.001		19.6 (17.5–22.5)	< 0.001		23.4 (21.4–27.2)	0.004
Sex									
Female	41 (85.4%)	82 (85.4%)			14 (73.7%)			14 (93.1%)	
Male	7 (14.6%)	14 (14.6%)			5 (26.3%)			2 (6.9%)	
Glucose, mg/dL	148 (118–184)	91 (84–103)	< 0.001		147 (112–189)	< 0.001		158 (120–184)	0.899
Creatinine, mg/dL	0.60 (0.52–0.78)	0.68 (0.59–0.77)	0.105		0.56 (0.5–0.8)	0.128		0.60 (0.53–0.82)	0.527
AST, IU/L	26 (17–43)	18 (15–23)	< 0.001		29 (17–46)	0.003		24 (17–31)	0.212
ALT, IU/L	25 (18–45)	17 (13–22)	< 0.001		30 (24–74)	< 0.001		22 (17–34)	0.025
Total cholesterol, mg/dL	208 (157–254)	211 (182–244)	0.468		160 (137–230)	0.005		229 (186–290)	0.013
Triglyceride, mg/dL	529 (305–917)	125 (92–166)	< 0.001		690 (406–921)	< 0.001		439 (289–930)	0.297
HDL cholesterol, mg/dL	31 (22–36)	52 (45–63)	< 0.001		28 (21–33)	< 0.001		33 (26–41)	0.018
LDL cholesterol, mg/dL	102 (71–128)	128 (107–161)	< 0.001		88 (69–109)	< 0.001		108 (72–153)	0.081
Leptin, ng/mL	0.9 (0.3–3.6)	NA	NA		0.3 (0.1–0.7)	NA		3.4 (1.3–8.2)	< 0.001
Adiponectin, ug/mL	4.5 (1.0–9.9)	NA	NA		1.1 (0.5–5.3)	NA		8.5 (2.9–13.7)	0.003
Aortomesenteric distance, mm	10.9 (6.6–16.1)	11.3 (8.4–15.5)	0.587		8.1 (6.0–10.8)	0.016		13.0 (8.8–18.1)	0.023
TF-SMA distance, mm	4.5 (2.3–22.2)	30.3 (13.9–46.6)	< 0.001		2.0 (1.5–3.5)	< 0.001		18.8 (4.4–42.2)	< 0.001
TF-RR distance, mm	20.0 (2.6–36.7)	29.7 (23.1–36.7)	< 0.001		1.3 (0.0–5.3)	< 0.001		33.7 (21.6–42.1)	< 0.001

Data are expressed as median (Q1–Q3). The Mann-Whitney U-test was used to calculate *P* values for continuous parameters. A *P* value of 0.05 or lower was considered statistically significant. Abbreviations: BMI, body mass index; ALT, alanine aminotransferase; AST, aspartate aminotransferase; HDL, high-density lipoprotein; LDL, low-density lipoprotein; FPLD, familial partial lipodystrophy; CGL, congenital generalized lipodystrophy; TF-SMA, distance between transversalis facia and the superior mesenteric artery, representing mesenteric fat tissue thickness; TF-RR, the distance from the end of the right renal lower pole to the transversalis facia, representing retroperitoneal fat tissue thickness; NA, not applicable

**Table 2:** Pathogenic variants detected in patients with CGL and FPLD

Variant	*n* = 48
**CGL**	19
***AGPAT2***	12
c.662–2 A > C, IVS5-2 A > C/ c.662–2 A > C, IVS5-2 A > C	1
c.144 C > A, p.(C48X)/ c.144 C > A, p.(C48X)	4
c.538_539delGA, p.(D180PfsX5)/ c.538_539delGA, p.(D180PfsX5)	1
c.514G > A, p.(E172K)/ c.514G > A, p.(E172K)	1
c.202 C > T, p.(R68X)/ c.202 C > T, p.(R68X)	2
c.685G > T, p.(E229X)/ c.685G > T, p.(E229X)	3
***BSCL2***	**4**
c.630 + 1G > A, IVS4 + 1G > A/ c.630 + 1G > A, IVS4 + 1G > A	2
c.280 C > T, p.(Q94X)/ c.280 C > T, p.(Q94X)	2
***CAVIN1***	**2**
c.481_482insGTGA, p.(K161SfsX51)/ c.481_482insGTGA, p.(K161SfsX51)	1
c.259 C > T, p.(Q87X)/ c.259 C > T, p.(Q87X)	1
***LMNA***	**1**
c.1745G > A, p. (R582H)/ c.1745G > A, p. (R582H)	1
**FPLD**	**29**
***LMNA***	**13**
c.139G > A, p.(D47N)/ wild type	1
c.14,565 A > G, p.(K486E)/ wild type	1
c.916 C > G, p.(L306V)/ wild type	1
c.1445G > A, p.(R482Q)/ wild type	2
c.1444 C > T, p.(R482W)/ wild type	6
c.1745G > A, p.(R582H)/ wild type	1
c.1583 C > T, p.(T528M)/ wild type	1
***PPARG***	**2**
c.1346 A > T, p.(H477L)/ wild type	1
c.452 A > G, p.(Y151C)/ wild type	1
**No variant detected**	**14**

Abbreviations: CGL, congenital generalized lipodystrophy; FPLD, familial partial lipodystrophy; *AGPAT2*, 1-acylglycerol-3-phosphate O-acyltransferase 2; *BSCL2*, Seipin; *CAVIN1*, caveolae associated protein 1; *LMNA*, lamin A/C

Among 29 patients with FPLD, 12 had typical Dunnigan syndrome due to heterozygous *LMNA* variants (Table 2). Partial lipodystrophy was characterized by fat loss primarily affecting the limbs with preservation/accumulation of fat in the upper portion of the body and/or face and neck in these patients. Fat loss was more severe in one patient with a pathogenic variant in *LMNA* c.1456 A > G, p.(K486E). This patient previously presented with generalized fat loss and coinciding systemic lymphoma [15]. Peroxisome proliferator-activated receptor gamma (*PPARG*) variants c.452 A > G, p.(Y151C) and c.1346 A > T, p.(H477L) were observed in two individuals. A slight fat loss affecting their limbs was noted in these two patients.

Of the remaining 14 patients with FPLD, seven exhibited phenotypic characteristics of the Kobberling variant (FPLD1). Genetic studies are typically unable to assist with the diagnosis of patients with FPLD1. Consequently, FPLD1 was diagnosed based on clinical features, including metabolic abnormalities, truncal obesity, fat loss predominantly affecting the extremities, and the presence of a palpable “ledge” between lipodystrophic and non-lipodystrophic areas. Furthermore, radiological studies, including whole-body MRI, were conducted to demonstrate fat loss or accumulation in specific body areas [16].

On the other hand, no pathogenic variant was identified in the genes currently associated with FPLD in the remaining seven patients with a phenotype indicating Mendelian FPLD (FPLDX). Historically, Sanger sequencing was used to study patients with lipodystrophy at our center. Typically, our FPLD genetic testing approach started with *LMNA* followed by *PPARG* sequencing. If these two genes did not yield disease-causing variants, Sanger sequencing was performed for other FPLD genes on a case-by-case basis based on phenotype. Over the past 8 years, our algorithm has shifted to the use of a genetic panel consisting of nine lipodystrophy genes.

Median glucose, aspartate aminotransferase (AST), alanine transaminase (ALT), and triglyceride levels were higher (*P* < .001, *P* = .001, *P* < .001, and *P* < .001, respectively), whereas the median high-density lipoprotein (HDL) levels were lower in patients with lipodystrophy than in controls (*P* < .001) (Table 1). Patients with GL were younger and had a lower BMI than the control group (*P* < .001). Conversely, glucose, AST, ALT, triglyceride, and LDL levels were higher in patients with GL than in the control group. Patients with FPLD were older and had a higher BMI than those with GL (*P* < .001 and *P* = .004, respectively). Although glucose, AST, triglyceride, and LDL levels were similar in both groups, patients with FPLD had higher levels of total cholesterol, HDL cholesterol, leptin, and adiponectin, and lower levels of ALT than patients with GL (Table 1). Among subtypes of FPLD, patients with FPLD1 had higher levels of leptin (*P* = .009) and lower levels of triglyceride (*P* = .036) compared to patients with FPLD2 (Table 3).

**Table 3:** Baseline characteristics and aortomesenteric distance among subtypes of patients with familial partial lipodystrophy

	FPLD 1 (*n* = 7)	FPLD 2 (*n* = 13)	*P* value
Age, years	40 (33–47)	46 (38–56)	0.721
BMI, kg/m^2^	26.7 (23.2–29.7)	23.4 (21.8–27.2)	0.284
Sex			
Female	6 (%85.7)	12 (%92.3)	
Male	1 (%14.3)	1 (%7.7)	
Glucose, mg/dL	165 (122–184)	181 (101–197)	0.751
Creatinine, mg/dL	0.52 (0.48–0.60)	0.71 (0.57–0.98)	0.039
AST, IU/L	20 (17–55)	17 (15–29)	0.327
ALT, IU/L	32 (18–54)	19 (15–24)	0.142
Total cholesterol, mg/dL	213 (146–289)	229 (186–273)	0.451
Triglyceride, mg/dL	324 (195–346)	660 (321–1069)	0.036
HDL cholesterol, mg/dL	37 (31–41)	31 (23–38)	0.362
LDL cholesterol, mg/dL	100 (56–128)	129 (88–156)	0.285
Leptin, ng/mL	11.5 (10.6–11.5)	1.8 (1.0–5.5)	0.009
Adiponectin, ug/mL	13.7 (4.2–13.7)	8.5 (3.6–10.0)	0.405
Aortomesenteric distance, mm	17.6 (13.0–20.0)	14.0 (10.9–18.4)	0.267
TF-SMA distance, mm	47.5 (37.6–60.0)	8.0 (4.2–29.1)	0.013
TF-RR distance, mm	41.8 (34.4–48.0)	27.0 (20.0–39.5)	0.043

Data are expressed as median (Q1–Q3). The Mann-Whitney U-test was used to calculate *P* values for continuous parameters. A *P* value of 0.05 or lower was considered statistically significant. Abbreviations: BMI, body mass index; ALT, alanine aminotransferase; AST, aspartate aminotransferase; HDL, high-density lipoprotein; LDL, low-density lipoprotein; FPLD, familial partial lipodystrophy; TF-SMA, distance between transversalis facia and the superior mesenteric artery, representing mesenteric fat tissue thickness; TF-RR, the distance from the end of the right renal lower pole to the transversalis facia, representing retroperitoneal fat tissue thickness

**Fig. 1:**
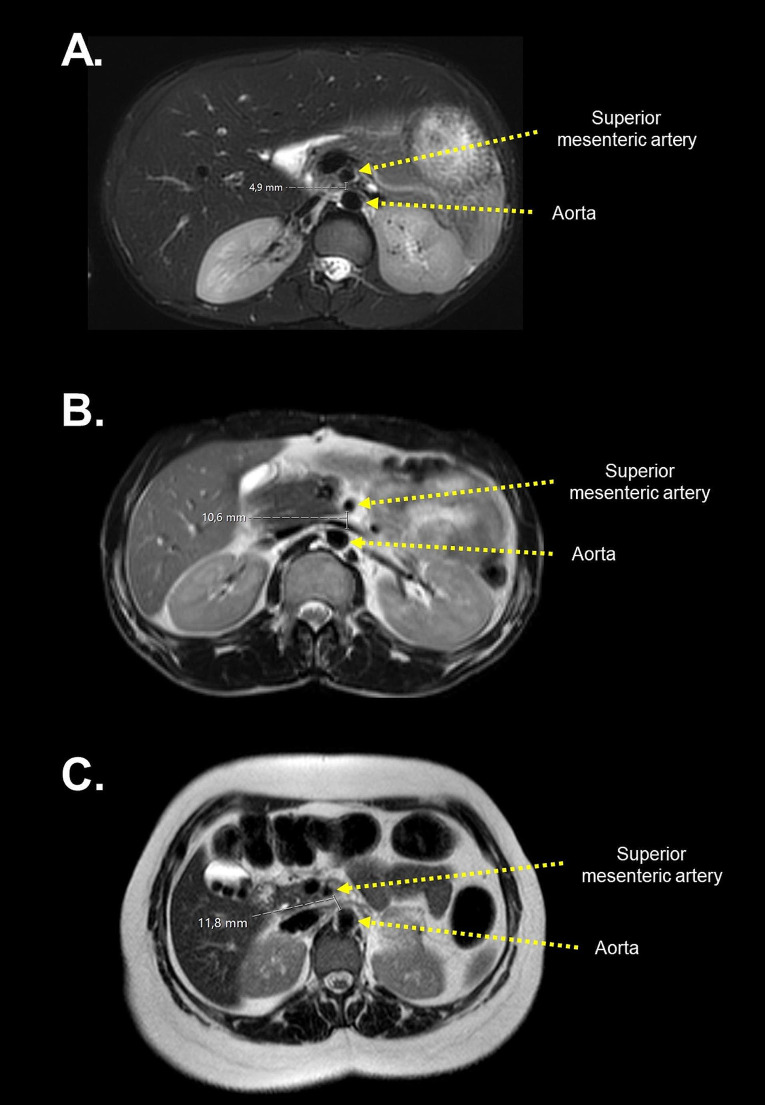
MR images showing aortomesenteric distance in healthy controls and patients with subtypes of genetic lipodystrophies (T1-weighted axial image at the level of left renal vein). The AOM distance was 4.9 mm in a 31-year-old female patient with CGL due to an *AGPAT2* pathogenic variant (**A**), 10.6 mm in a 32-year-old female patient with FPLD due to an *LMNA* pathogenic variant (**B**), and 11.8 mm in a 34-year-old female healthy control (**C**)

Median AOM distance was 10.9 (IQR: 6.6–16.1) mm in patients with lipodystrophy and 11.3 (IQR: 8.4–15.5) mm in the control group (*P* = .587). Patients with CGL had a lower AOM distance (8.1, IQR, 6.0–10.8 mm) compared with controls (11.3, IQR, 8.4–15.5 mm; *P* = .016) and patients with FPLD (vs. 13.0, IQR, 8.8–18.1 mm; *P* = .023). The AOM distance was numerically higher in patients with FPLD1 than in those with FPLD2, but this was not statistically significant. Figure 1 shows representative AOM distance measurements in T1-weighted MR scans from patients with two subtypes of lipodystrophy and healthy controls.

**Fig. 2:**
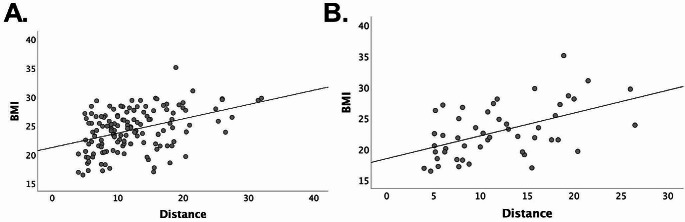
Positive correlation between BMI and AOM distance in all subjects (**A**) and in patients with lipodystrophy (**B**)

BMI was positively correlated with the AOM distance, both in the overall sample (*r* = .397, *P* < .001) and in patients with lipodystrophy (*r* = .505, *P* < .001) (Fig. 2). BMI was found to be a significant predictor of the AOM distance in multivariate linear regression analysis including an age- and sex-adjusted model (beta = 0.537, 95% confidence interval [CI]:0.277–0.798, *P* < .001). No correlation was observed between height and AOM distance in all subjects (*P* = .976), among patients with lipodystrophy (*P* = .319), or in the control group (*P* = .364).

**Fig. 3:**
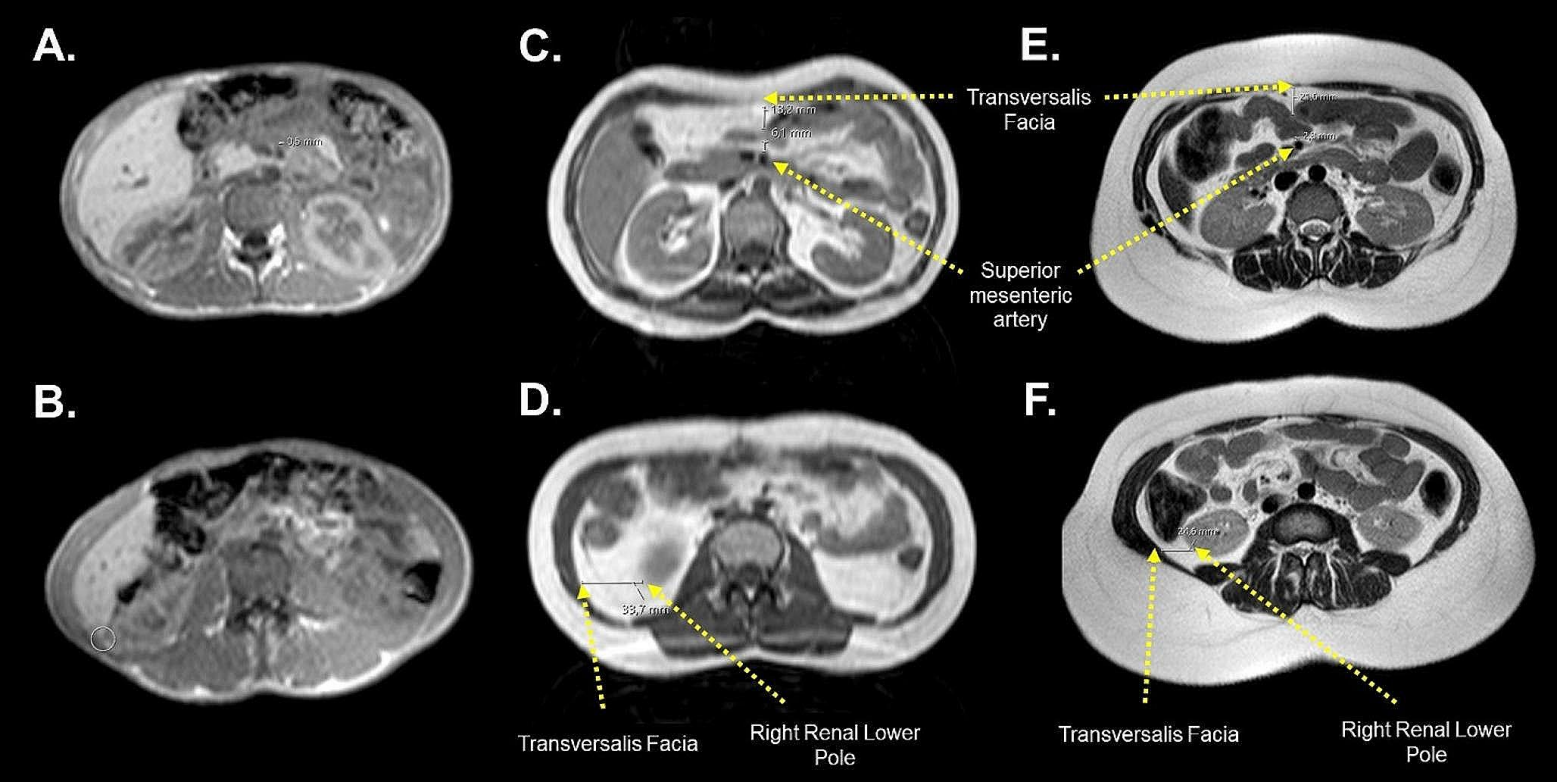
MR images showing the distance between the transversalis facia and the superior mesenteric artery (TF-SMA) at the level of the umbilicus which estimates mesenteric fat tissue thickness in healthy controls and patients with subtypes of genetic lipodystrophies (T1-weighted axial image). Retroperitoneal fat tissue thickness was estimated by measuring the distance from the end of the right renal lower pole to the transversalis facia (TF-RR). Mesenteric (**A**) and retroperitoneal (**B**) fat tissues were almost completely lost in a 29-year-old female patient with CGL due to an *AGPAT2* pathogenic variant. The TF-SMA and TF-RR distances were 19.3 mm (**C**) and 33.7 mm (**D**) in a 33-year-old female patient with FPLD lacking a pathogenic variant, and 23.4 mm (**E**) and 24.6 mm (**F**) in a 34-year-old female healthy control, respectively

The TF-SMA and TF-RR distances, representing mesenteric and retroperitoneal adipose tissues, respectively, were significantly lower in patients with CGL than in patients with FPLD and controls (Table 1). Although patients with FPLD had a lower TF-SMA distance than the controls (*P* = .016), the TF-RR distance was similar between the two groups. Among patients with FPLD subtypes, those with FPLD1 had higher TF-SMA and TF-RR distances compared to those with FPLD2 (Table 3). BMI and AOM distance were positively correlated with TF-SMA (*r* = .429, *P* < .001 and *r* = .493, *P* < .001, respectively) and TF-RR (*r* = .488, *P* < .001 and *r* = .503, *P* < .001, respectively) distances in all subjects. Similar correlations were found when patients with lipodystrophy were analyzed separately. BMI and AOM distance were positively correlated with TF-SMA (*r* = .633, *P* < .001 and *r* = .545, *P* < .001, respectively) and TF-RR (*r* = .581, *P* < .001 and *r* = .490, *P* < .001, respectively) distances in patients with lipodystrophy. Representative mesenteric and retroperitoneal fat measurements on T1-weighted MR scans from patients with two subtypes of lipodystrophy and healthy controls are shown in Fig. 3.

Leptin levels were lower in patients with CGL (0.3 ng/mL, IQR, 0.1–0.7 ng/mL) than in patients with FPLD (3.4 ng/mL, IQR, 1.3–8.2 ng/mL; *P* < .001). Leptin was positively correlated with AOM distance in patients with lipodystrophy (*r* = .513, *P* < .001). A positive correlation was observed between leptin levels and AOM distance in patients with FPLD (*r* = .551, *P* = .005). Leptin levels were positively correlated with TF-SMA and TF-RR distances in patients with lipodystrophy (*r* = .717, *P* < .001 and *r* = .721, *P* < .001, respectively). In addition, adiponectin levels were lower in patients with CGL than in those with FPLD (*P* = .003). Adinopectin was positively correlated with leptin (*r* = .554, *P* < .001), but there was no correlation between AOM distance and adiponectin.

Of the 19 patients with CGL, 12 (63%) had an AOM distance of less than 10 mm, a risk factor that may predispose to SMA syndrome development. Among these patients, four were admitted to the emergency room or hospital with abdominal pain or GI symptoms: two with confirmed pancreatitis, one diagnosed with intestinal perforation, and one patient had an undetermined etiology of abdominal pain. Our medical chart review identified a patient with symptoms suggestive of SMA syndrome in addition to recurrent pancreatitis. A 37-year-old female patient with CGL due to an *APGAT2* variant (BMI: 16.9 kg/m^2^) underwent seven hospitalizations for abdominal pain, all of which were attributed to episodes of pancreatitis secondary to hypertriglyceridemia. The patient was initiated on metreleptin at a dose of 5 mg/day. The patient remained on the same dose as we observed continuous desirable benefits from the treatment with no significant side effects. Her severe abdominal pain episodes were resolved with metreleptin treatment, and thereby, no further hospitalization was required.

**Fig. 4:**
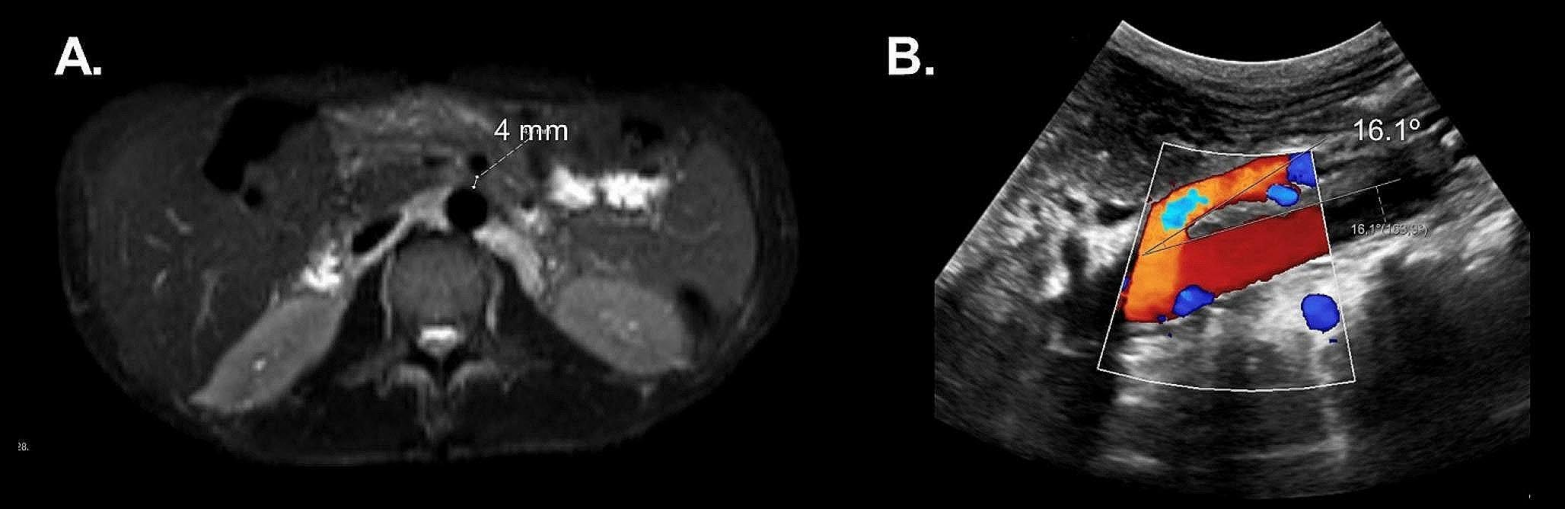
MR images and Doppler ultrasonography of a 37-year-old woman with CGL due to an *AGPAT2* pathogenic variant. (**A**) Abdominal MR images showing aortomesenteric distance of 4 mm (fat-saturated T1-weighted axial image at the level of left renal vein). (**B**) Doppler Ultrasonography showing the aortomesenteric angle of 16.1º

Upon reviewing her medical charts, amylase and lipase levels and CT assessments were incompatible with acute pancreatitis in at least two of these hospitalizations. During symptom examinations focusing on periods outside hospitalization episodes, the patient reported chronic abdominal pain for years, describing postprandial epigastric pain accompanied by nausea. Vomiting would occasionally follow abdominal pain, typically radiating from the midline of the abdomen to the waist. The CT could not measure the AOM angle because of low body weight. Transabdominal Doppler USG was performed, which revealed an AOM angle of 16.1º, indicating a severely narrowed AOM angle that can lead to SMA syndrome (Fig. 4).

One additional patient had an undetermined etiology of abdominal pain, with a 28º AOM angle measured in CT images from her medical records. Unfortunately, further symptom reviews or diagnostic testing could not be conducted because the patient died.

Eight of 29 (27%) patients with FPLD had an AOM distance of 10 mm. Among them, two were hospitalized several times with clinical symptoms of acute pancreatitis due to severe hypertriglyceridemia. Notably, no other patients with FPLD reported symptoms suggestive of SMA syndrome or displayed an AOM angle of < 25º on available CT images in the medical charts.

## Discussion

Abdominal pain is a common symptom of lipodystrophy. Patients with lipodystrophy develop severe metabolic problems, causing a significant disease burden [17, 18]. The disease is associated with severe hypertriglyceridemia, a lipid abnormality that can lead to acute pancreatitis [19]. Many patients with lipodystrophy, when admitted with abdominal pain, are typically diagnosed with acute pancreatitis [20, 21]. However, several other etiologies can cause abdominal pain in patients with lipodystrophy, with abdominal pain due to intestinal dysmotility being a typical example in patients with CGL4 [22]. Although intestinal dysmotility often manifests as chronic, low-grade abdominal pain in CGL4, it can also lead to complications such as intestinal perforation, presenting with a sudden onset of severe abdominal pain that can be life-threatening if not promptly diagnosed [14, 23].

Theoretically, the loss of mesenteric fat in lipodystrophy could trigger the SMA syndrome development. Nevertheless, no previous case reports have described the SMA syndrome development in lipodystrophy. This could be influenced by the difficulty in diagnosing SMA syndrome, requiring confirmatory testing through comprehensive radiographic studies. Our study presents the first attempt to identify the risk of developing SMA syndrome in lipodystrophy. Although we could not formally diagnose a case of SMA syndrome, apart from a single instance in a patient with CGL exhibiting symptoms and a very narrow AOM angle aligning with SMA syndrome, most patients with CGL had a shortened AOM distance underscoring the potential risk of SMA syndrome development in this rare patient population.

A previous study has demonstrated that the distance between the SMA and the aorta shortens in those with SMA syndrome [24]. In addition, the AOM distance is strongly correlated with clinical symptoms of SMA syndrome [5]. Measuring the AOM distance and angle proves to be a useful approach to detecting SMA syndrome in patients with clinical findings, although further radiological assessments may be necessary for confirmation [24, 25]. Normal AOM angle and distance values typically range between 45° and 60° and 10–20 mm, respectively [3, 5]. An AOM angle of < 25° appears to be a reliable diagnostic indicator, especially when accompanied by a reduction in the AOM distance to 8 mm [5, 26].

Our results confirmed a shortened AOM distance in patients with CGL, whereas the AOM distance was similar between patients with FPLD and controls. Among the 19 patients with CGL, twelve had an AOM distance shorter than 10 mm, and nine had an AOM distance shorter than 8 mm. Patients with CGL typically experience near-total fat loss affecting mesenteric fat, whereas visceral fat is usually preserved or even accumulated in patients with FPLD [27, 28]. BMI emerged as a significant predictor of AOM distance, consistent with findings in non-lipodystrophic subjects [29]. As expected, BMI was lower in patients with CGL than in those with FPLD and controls. In addition, serum leptin levels were correlated with AOM distance. Patients with CGL typically have very low or undetectable leptin levels [18].

To further study the association of visceral fat with AOM distance, we measured different intra-abdominal sites representing mesenteric and retroperitoneal adipose tissue. As expected, and in line with previous studies, patients with GL had little or no mesenteric and retroperitoneal adipose tissue [11, 30]. AOM distance was positively correlated with both measurements.

Our study lacks the capacity for the formal diagnosis of SMA syndrome which would require comprehensive radiological assessments. However, medical charts were reviewed to identify any indications of SMA syndrome and to evaluate symptoms that could be associated with duodenal outlet obstruction. During our review, a patient with CGL exhibited a short AOM distance and a significantly narrow AOM angle and reported gastrointestinal symptoms such as chronic abdominal pain, nausea, and occasional vomiting, in addition to several episodes of confirmed pancreatitis, which were mostly resolved after the metreleptin treatment. It is widely recognized that leptin replacement improves hypertriglyceridemia in lipodystrophy [16], thereby preventing further episodes of pancreatitis [30]. Leptin replacement therapy also regulates appetite and reduces food consumption [31, 32]. However, whether the reduced food intake in our patient contributed to the improvement of duodenal outlet obstruction, possibly due to smaller amounts of food passing through a narrow transit point, remains unknown.

Our study has several limitations. First, our standard MRI protocol for fat distribution lacks contrast-enhanced T1-weighted sagittal plane images, which are crucial for efficiently assessing the AOM angle. Consequently, the AOM distance was systematically measured in all patients and controls using MR images, whereas AOM angle measurements were completed only in a select patient population where abdominal CT images were available. Furthermore, Doppler ultrasonography was used to measure the AOM angle in one patient who exhibited symptoms indicative of SMA syndrome. Second, our study adopted a retrospective design and included a small number of male patients. Third, our findings require confirmation in different populations with diverse ethnic backgrounds. Finally, the results of our study should be validated in a broader cohort of patients with clinically diagnosed SMA syndrome.

In conclusion, our study indicates that the loss of mesenteric fat in CGL results in a shortened AOM distance, potentially increasing the risk of SMA syndrome development. Abdominal pain often arises from episodes of acute pancreatitis due to extremely high triglyceride levels in lipodystrophy; however, it can be caused by various reasons. Our study contributes by adding SMA syndrome to this list, expanding the differential diagnosis of abdominal pain in CGL, particularly when initial tests do not indicate acute pancreatitis.

## Data Availability

The datasets produced and/or analyzed in this study may not be publicly accessible. However, they can be obtained from the corresponding author upon reasonable request.
